# Early differences in lassitude predicts outcomes in Stanford Neuromodulation Therapy for difficult to treat depression

**DOI:** 10.1038/s44184-024-00099-2

**Published:** 2024-10-28

**Authors:** David Benrimoh, Azeezat Azeez, Jean-Marie Batail, Xiaoqian Xiao, Derrick Buchanan, Igor D. Bandeira, Andrew Geoly, Yaakov Keynan, Ian H. Kratter, Nolan R. Williams

**Affiliations:** 1 Brain Stimulation Lab, Department of Psychiatry and Behavioral Sciences, Stanford University, Palo Alto, CA USA; 2 McGill University, Department of Psychiatry, Montreal, QC Canada; 3 Pôle Hospitalo-Universitaire de Psychiatrie Adulte, Centre Hospitalier Guillaume Régnier, Rennes, France; 4 Department of Radiology, Stanford University, Stanford, CA USA

**Keywords:** Neuroscience, Biomarkers, Health care, Medical research

## Abstract

Stanford Neuromodulation Therapy (SNT), has recently shown rapid efficacy in difficult to treat (DTT) depression. We conducted an exploratory analysis of individual symptom improvements during treatment, correlated with fMRI, to investigate this rapid improvement in 23 DTT participants from an SNT RCT (12 active, 11 sham). Montgomery–Åsberg Depression Rating Scale item 7 (Lassitude) was the earliest to show improvements between active and sham, as early as treatment day 2. Lassitude score at treatment day 3 was predictive of response at 4 weeks post-treatment and response immediately after treatment. Participants with lower lassitude scores at treatment day 3 had different patterns of sgACC functional connectivity compared to participants with higher scores in both baseline and post-treatment minus baseline analyses. Further work will aim to first replicate these preliminary findings, and then to extend these findings and examine how SNT may affect lassitude and behavioral activation early in treatment.

## Introduction

Major depressive disorder (MDD) is the leading global cause of disability, affecting one in nine people in the U.S. over the course of their lives, and generates costs upwards of $200B USD annually^1^. In addition to being a common mental disorder, MDD is also difficult to treat successfully. Over two-thirds of patients will not respond to their first pharmacological treatment, and at least one-third will continue to experience symptoms after four treatment trials^2^. Patients who do not respond to several adequate treatments may be classified as having difficult to treat depression (DTT), also known as treatment-resistant depression, a status with several definitions^3^. Unfortunately, patients who do not respond to a first or second treatment tend to have lower rates of response to later treatments, and some patients with DTT depression can also require costly and prolonged hospitalizations^4^. Accordingly, there is a specific need for novel interventions focusing on DTT depression.

One modality which can effectively treat DTT depression without adding to patient medication burden, and with minimal side effects, is repetitive transcranial magnetic stimulation (rTMS)^3^. rTMS has level one evidence for MDD and was approved by the United States Food and Drug Administration (FDA) in 2008 as a treatment option for patients who have not responded to at least one antidepressant^5^. While most rTMS protocols require up to six weeks to be effective, the Stanford Neuromodulation Therapy (SNT) protocol was developed^6^ to deliver treatment in an accelerated manner, taking only five days. This treatment protocol utilizes intermittent theta burst stimulation (iTBS) targeted at the left dorsolateral prefrontal cortex (LDLPFC) and delivers treatments in an accelerated manner: 50 iTBS sessions over 5 days (10 sessions per day of 1800 pulses spaced one hour apart). In addition to its accelerated time frame, it personalizes treatment by using neuronavigation to allow a precise placement of the rTMS coil; specifically over the region of the LDLPFC which, for each patient, demonstrates the highest anti-correlation on resting state functional magnetic resonance imaging (rsfMRI) with the subgenual anterior cingulate cortex (sgACC). This focus on LDLPFC-sgACC anticorrelation is due to evidence that LDLPFC-rTMS efficacy is mediated through the downregulation of sgACC activity by increased activity in the LDLPFC caused by the rTMS^7,8^.

In a recent small, randomized, double-blind, sham-controlled trial (RCT), active SNT was found to be more effective than sham in patients with DTT depression (a 62.0% mean percent reduction at one week post treatment and a 52.5% mean percent reduction at 4 weeks post treatment from baseline MADRS scores in the active treatment group compared to 14.3% and 11.1% in the sham group for the same time points)^9^. These results cohere with those from a previous open label study^6^. Although these results are positive and encouraging, questions remain about what mechanism accounts for this rapid antidepressant effect. Traditional rTMS protocols, antidepressants, and psychotherapies generally require 4–8 weeks of treatment for a full antidepressant treatment effect^10^. Examining why SNT can produce benefit after 5 days of treatment may be useful in the search for novel mechanisms or biomarkers discovered that may serve to augment efforts aimed at generating novel treatments for DTT depression.

In this paper we engage in an exploratory re-analysis of data from the SNT RCT^9^ with the objective of generating hypotheses regarding potential mechanisms and biomarkers relating to the rapid effectiveness of SNT. In particular, we were interested in identifying early changes in individual symptoms during treatment, as we hypothesize that the symptoms which show the earliest changes might be indicative of neurobiological mechanisms underlying rapid response to SNT. In order to accomplish this, we examine two sources of data. The first is daily individual symptoms, measured using the Montgomery-Asberg Depression Rating Scale (MADRS), which we examined to determine which symptoms showed the earliest change during treatment. The second is functional connectivity data from fMRI scans pre- and post-treatment. We correlated this functional connectivity data with early symptom changes, in order to identify putative biomarkers for investigation in larger studies.

## Methods

### Sample

We re-analyzed data from a previous SNT RCT (please see ref. ^9^ for detailed methods and patient population). 23 participants had daily symptom data available; as examining individual symptom change during the 5 day SNT protocol was our main priority, these were the participants included in the analysis. Twelve of the participants were randomized to the active condition and 11 to the sham condition. The study was carried out between March 2017 and December 2019 at the Department of Psychiatry and Behavioral Sciences at Stanford University. Key inclusion criteria included moderate-to-severe MDD (≥20 on the Montgomery-Åsberg Depression Rating Scale (MADRS)^11^, 22–80 years of age, stable medication regimen (if any) for at least 4 weeks, and to maintain this regimen during the study (both during acute treatment and follow-up). Exclusion criteria included primary psychiatric diagnoses other than MDD, conditions which increase rTMS risk (see supplementary of^9^ for a detailed list), previous exposure to rTMS, previous non-response to ECT, and a history of psychosurgery. While participants were required to have total MADRS severities of at least 20 points, there were no requirements regarding the severities of individual symptom items. All participants provided informed consent for the study prior to participation, and all procedures were approved by the Stanford University School of Medicine Institutional Review Board. Clinical Trials registry for the original study NCT03068715.

### Assessments

The main assessment of interest for this analysis was the MADRS. The MADRS is considered at baseline, at 1, 2 and 4 weeks post treatment, as well as daily during treatment. All MADRS assessments at baseline and at 1-, 2-, and 4-weeks post treatment were completed by expert raters. Those completed daily during treatment days were completed by psychology interns for monitoring purposes. No other daily ratings were reliably available for the analysis.

### Statistical analysis for daily symptom data

The first question we sought to answer was: when comparing sham to active groups, which of the individual symptoms on the MADRS changed first during acute treatment, and in what order do symptoms change? In order to answer this, we conducted an ANOVA for each of the 10 MADRS items on each of the 5 days of treatment, comparing active and sham groups. We looked for the individual symptoms which showed the earliest separation between active and sham groups. All subjects had complete data for daily ratings and were included in the ANOVA.

After identifying the symptom with the earliest difference between groups (as discussed below, this was MADRS item 7, Lassitude), we conducted a repeated measures general linear model (GLM) to confirm that, over time, the value of the symptom was different between the active and sham groups; data was available for 19 patients for this analysis as 4 were missing data in post-treatment weeks. In the supplementary results, we present results for a GLM restricted to the first 5 days with complete data for all 23 patients and similar results.

Finally, we asked whether the value of MADRS item 7 early in treatment (on day 3) could predict treatment response at 4 weeks post-treatment and response immediately post-treatment (measured 3–4 days after the last treatment day). Two subjects were missing week 4 data, but had data for immediate followup. Neither of these patients were responders at immediate followup; in order to increase power, for these patients last-observation carried-forward was used in the regression analysis predicting response at 4 weeks. Response was defined as a 50% decrease from baseline MADRS total score. Remission was also examined (see supplementary material). We employed a binary logistic regression model with a 1000 sample bootstrap for these analyses. We controlled for the total baseline MADRS score in order to account for differences in baseline depression severity. Models were assessed by examining significance of individual parameters and comparison to a null model in terms of nagelkerke R2.

As this was an exploratory analysis, we did not correct for multiple comparisons. All analyses were conducted using SPSS. Alpha was set at 0.05. Greenhouse-Geisser corrections were used when sphericity could not be assumed.

### rsfMRI data acquisition

MRI safety of all participants was assessed prior to scanning. Scans were performed pre-treatment and post-treatment and included a structural T1 weighted image and an 8-min resting-state functional MRI acquisition. All images were acquired using a 3TGE Discovery MR750 scanner with a 32-channel head-neck imaging coil at the Center for Cognitive and Neurobiological Imaging at Stanford University. Additional acquisition parameters can be found in Cole et al. ^9^. All 23 patients had pre-treatment scans, therefore analysis focused on baseline scans was conducted for all patients; 21 patients had post-treatment scans, therefore analyses focused on pre-post changes were conducted for this subset.

### rsfMRI functional connectivity analysis

The preprocessing pipeline used is discussed in ref. ^12^. MRI data were preprocessed using FMRIPREP version 20.2.0 [RRID:SCR 016216], T1-weighted (T1w) volume was corrected for intensity non-uniformity using N4BiasFieldCorrection v2.1.0 and skull-stripped using antsBrainExtraction.sh v2.1.0 (using the OASIS template). Spatial normalization to the ICBM 152 Nonlinear Asymmetrical template version 2009c [RRID:SCR 008796] was performed through nonlinear registration with the antsRegistration tool of ANTs v2.1.0 [RRID:SCR 004757], using brain-extracted versions of both T1w volume and template. Brain tissue segmentation of cerebrospinal fluid (CSF), white-matter (WM) and gray-matter (GM) was performed on the brain-extracted T1w using fast (FSL v5.0.9, RRID:SCR 002823). The resting state data were motion-corrected using mcflirt (FSL v5.0.9), followed by co-registration to the T1w using flirt (FSL). Motion correcting transformations, BOLD-to-T1w transformation and T1w-to-template (MNI) warp were concatenated and applied in a single step using antsApplyTransforms (ANTs v2.1.0) using Lanczos interpolation. Framewise displacement was calculated for each functional run using the implementation of Nipype. All volumes with framewise displacement (FD) greater than 0.5 mm were excluded. ICA-based Automatic Removal Of Motion Artifacts (AROMA) was used to generate aggressive noise regressors and create a variant of data that is non-aggressively denoised. The average signal within anatomically-derived eroded CSF and WM masks were included as confounder regressors. Data were spatially smoothed (6-mm-full-width, half-maximal Gaussian kernel) and temporal bandpass filtered (0.01–0.1 Hz). Finally, all data were detrended using Nilearn.

A parcel-based Region of Interest (ROI) analysis approach was taken and incorporated 120 total ROIs in a custom atlas parcellation. This parcellation included 100 cortical ROIs from the 7 network Schaefer brain parcellation^13^, 6 bilateral subregions of the amygdala from the Juelich histological atlas^14^, 6 bilateral subregions (executive, sensorimotor and limbic) of the striatum, bilateral thalami and hippocampi from the Harvard-Oxford cortical and subcortical structural atlases^15^, and finally bilateral sgACC and DLPFC ROIs from the Brodmann-Yale atlas. As such, the binomial coefficient (unique combinations) of 120 regions is 7140. Given our small sample size, we chose to focus on the FC for one region of interest in order to limit the potential for the detection of spurious connectivity findings. We considered two candidate regions: the LDLPFC - the stimulation site - and the sgACC. The sgACC was considered because the SNT target is defined based on anticorrelation with sgACC. As the clinical efficacy of LDLPFC rTMS correlates with FC of the stimulation target with the sgACC^16^, and SNT depends on careful neuronavigation to an LDLPFC target defined for each individual based on FC with the sgACC, we hypothesized that changes in sgACC FC may be most informative to examine given our objective of probing potential mechanisms of rapid response to SNT. In addition, the sgACC is a region implicated in many studies as part of the network underlying depressive symptoms and which is modulated during response to treatment^17^. As such, we chose to examine only connections between the sgACC and other brain regions. Consequently, for each (left and right) hemisphere of the sgACC there are 118 FC pairs to other ROIs and then one additional interhemispheric sgACC FC pair. In total this amounts to 237 FC pairs in which the sgACC was a member. Note that we considered both the left and right sgACC; while the rTMS target is the left DLPFC, several studies have shown that clinical efficacy correlates with FC with targets localized on the right or midline sgACC^16,18^.

For the resting state functional connectivity analyses, we sought to examine differences in FC between groups with high and low scores on the symptom which changed earliest during treatment. As such, we split patients into two groups: those with a high or low score on the symptom which was identified as differing between active and sham groups the earliest. The “low” group was defined as those with a score of 2 or less on the symptom measured at day 3, and the “high” group as those with a score of 3 or more (the cut-point was determined by examining the distribution of the symptom and selecting the median value). This left 15 patients in the “low” group (of which 4 were patients who received sham treatment) and 8 in the “high” group (of which 7 were patients who received sham treatment).

We conducted two analyses. In the first, we looked only at baseline functional connectivity. ANOVA was then used to examine FC differences across all sgACC connected regions between the “high” and “low” groups. The purpose of this analysis was to determine if there were any *predictive* pre-treatment biomarkers in rsfMRI which were related to early symptom differences which are predictive of outcome.

In the second analysis, we subtracted the functional connectivity values at 4 weeks post treatment from those at baseline to generate functional connectivity change values. ANOVA was then used to examine FC change differences across all sgACC connected regions between the “high” and “low” groups. The purpose of this analysis was to determine if there were any *response* biomarkers in rsfMRI which were indicative of differences between those with and without early low scores on a symptom which is predictive of outcome. Figures were generated using BrainNet viewer^19^.

Given the low power in our dataset and the fact that this was an exploratory analysis, significance was assessed at an alpha of 0.01 so as to minimize Type II errors.

## Results

### Sample characteristics

Age in years was not significantly different between the active and sham groups (mean active 49.3 (SD = 15.5); mean sham 52.8 (SD = 16.1); *F* = 0.29, *p* = 0.59). The overall mean age of the sample was 51 (SD = 15.52). Maudsley score (Fekadu et al., 2009) was not different between groups (active mean 8.75 (SD = 1.76), sham mean 9.18 (SD = 2.08); *F* = .289; *p* = 0.6). There were 16 males and 7 females in the sample, with 8 males in each group; the gender distribution was not significantly different between groups (*X2* = 0.1; *p* = 0.75).

### Early symptom differences between groups

We conducted a one-way ANOVA comparing active and sham groups on each of the individual items of the MADRS as well as the total score on each day of treatment. At baseline pre-treatment and on day 1, there were no between-group differences on any of the items. On day 2, the only item that was significantly different between groups was MADRS item 7 (Lassitude) (F = 4.94, *p* = 0.037); the active group had lower scores than the sham group. The same finding was observed on day 3 (F = 7.48, *p* = 0.012); and on day 4 (F = 13.54, *p* = 0.001). On day 5, a number of other significant findings emerged, with scores always being lower in the active group. The significantly different findings on individual items at day 5 were: MADRS item 1 (Apparent Sadness) (*F* = 4.91, *p* = 0.038); MADRS item 4 (Reduced Sleep) (F = 9.00, *p* = 0.007); MADRS item 7 (Lassitude) (*F* = 6.53, *p* = 0.018); and, MADRS item 8 (Inability to Feel) (F = 6.61, *p* = 0.018). This was also the first time point at which significant differences were noted in MADRS total score (F = 5.98, *p* = 0.023). Differences in symptoms on expert ratings immediately post-treatment, at 1 week and at 4 weeks post treatment are presented in the supplementary material. Table 1 provides data on MADRS item 7 scores at baseline and during the treatment days.

**Table 1:** MADRS Item 7 scores at baseline and each treatment day, presented with differences between active and sham as well as ANOVA results

Timepoint	Baseline	Treatment Day 1	Treatment Day 2	Treatment Day 3	Treatment Day 4	Treatment Day 5
MADRS Item 7 Sham - Mean (SD)	3.45 (1.04)	3.45 (1.13)	3.27 (0.91)	2.73 (0.91)	2.82 (0.98)	2.45 (1.7)
MADRS Item 7 Active - Mean (SD)	3.5 (1.24)	2.58 (1.56)	2.17 (1.4)	1.58 (1.08)	1.25 (1.06)	0.92 (1.17)
Difference, Sham- Active	−0.05	0.87	1.1	1.15	1.57	1.53
F value	0.01	2.31	4.94	7.48	13.54	6.53
*P* value	0.925	0.144	0.037*	0.012*	0.001**	0.018*
*n*=	23	23	23	23	23	23

*P < 0.05, **P < 0.01.

**Fig. 1:**
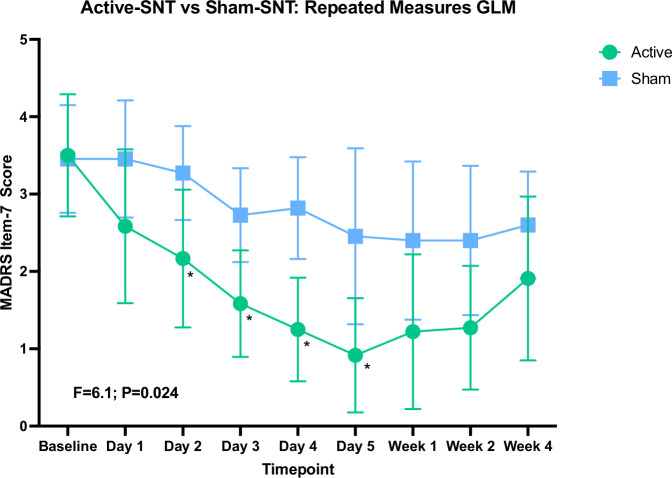
Trajectories of MADRS item 7 scores. Results shown for repeated measures GLM between-subjects main effect between active and sham groups. There was no significant group x time interaction. Figure generated using Prism. Day 1–Day 5 are treatment days; Week 1–Week 4 are weeks post-treatment. Data available for 19 subjects. Error bars represent 95% CI. * indicates significant difference between groups on scores for that timepoint (ANOVA, *p* ≤ 0.05).

To visualize lassitude trajectories and further investigate whether lassitude is different overall between the active and sham groups, we conducted a repeated measures GLM. As shown in Fig. 1, MADRS item 7 for all participants with a complete data set was significantly different between groups (main effect, between subjects F = 6.1; *p* = 0.024). There was also a main effect of time (F(2.9) 6.17, Greenhouse-Geisser corrected *p* = 0.001) but no time by group interaction (*p* = 0.28). Analysis limited to the baseline to treatment day 5 time points included all participants and revealed similar results (see supplementary material).

### Lassitude as a predictor of outcome

We next examined if MADRS item 7 early in treatment is predictive of treatment outcome. We chose to examine MADRS item 7 score at day 3 in the context of limited power because the differences between active and sham were more robust on day 3 than on day 2 (see supplementary material for prediction of outcome using score at day 2). 75% of active patients and 18% of controls were responders immediately after treatment; 58% of active patients and 9% of controls were responders at 4 weeks post treatment. A bootstrapped binary logistic regression model demonstrated that, when controlling for baseline MADRS total score, day 3 MADRS item 7 scores were predictive of treatment response at 4 weeks (model: *X2*(2) = 8.28; *p* = 0.016; lassitude (B = −1.55, *p* = 0.005, 95% CI: [−53.57, −0.69])). The model had a nagelkerke R2 of 0.42, and outperformed the null model. For response immediately post-treatment, MADRS item 7 score at day 3 was again predictive (model: *X2*(2) = 13.05; *p* = 0.02; lassitude (B = −2.3, *p* = 0.012, 95% CI: [−67.9, −1.2]); the model had a nagelkerke R2 of 0.58.

Additionally, we found that MADRS item 7 score at day 3 correlated with total MADRS score at week 4 post treatment (*r* = 0.74, *p* = 0.006), and that MADRS item 7 was different between responders and non-responders at week 4 post-treatment, even when correcting for MADRS total at baseline (see supplementary sections C and G).

Given that this analysis was exploratory and uncorrected, we sought to replicate it in another dataset. While no other SNT dataset with daily MADRS ratings was available, daily ratings using the HAMD-6 were available from our previous open label study of the SNT protocol^6^. Results of this bootstrapped logistic regression analysis, reported in the supplementary material, demonstrated that, controlling for baseline MADRS total score, HAMD-6 psychomotor retardation score (the available item most closely related to lassitude on the MADRS^20^) at day 3 was a predictor of response at 4 weeks post treatment (B = −23.2, *p* = 0.005; model Nagelkerke R2 = 0.35).

### rsfMRI differences pre-treatment

**Fig. 2:**
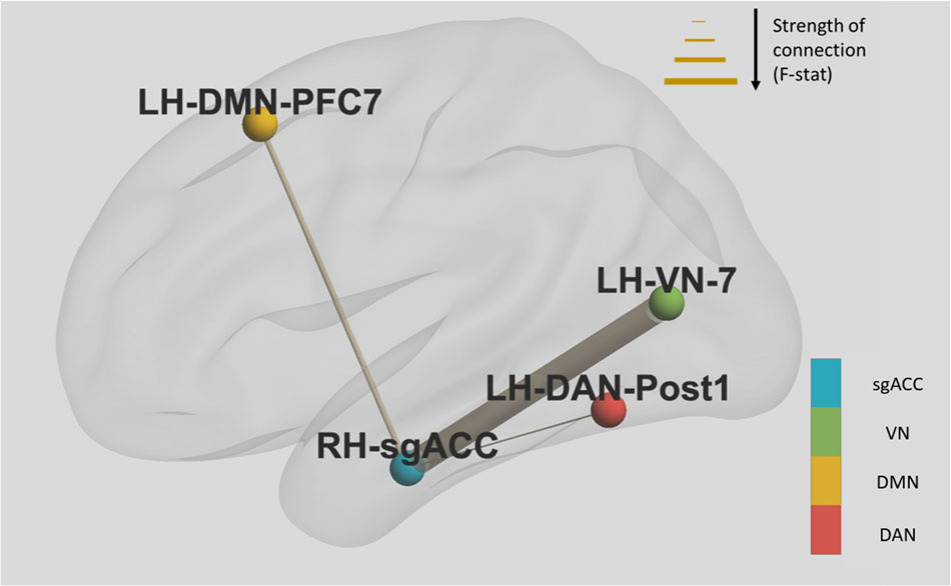
Pre-treatment resting state functional connectivity which differed between groups with high vs low lassitude score at treatment day 3. Thickness of connections between nodes is determined by the F-statistic. Directionality of connectivity was as follows: right sgACC to LH_VN_7 - patients in the low lassitude group showed reduced FC; right sgACC and LH_DAN_Post1- patients in the low group showed reduced FC; right sgACC and LH_DMN_PFC7- patients in the low group showed increased FC. Notes: dorsal attention network (DAN); visual network (VN); default mode network (DMN).

Three pretreatment FC pairs were found to be significantly different between groups which scored high and low on Lassitude at day 3. These pairs involved regions that were part of the dorsal attention network (DAN); visual network (VN); default mode network (DMN) and were: right sgACC to Left Hemisphere Visual Area 7 where patients in the low group showed reduced FC (F = 14.38, *p* = 0.001); right sgACC and Left Hemisphere Dorsal Attention Network Posterior area 1 where patients in the low group showed reduced FC (F = 9.17, *p* = 0.006); and right sgACC and Left Hemisphere Default Mode Prefrontal area 7 where patients in the low group showed increased FC (F = 10.06, *p* = 0.005). These connections are demonstrated in Fig. 2.

### rsfMRI differences post-treatment-pre-treatment

**Fig. 3:**
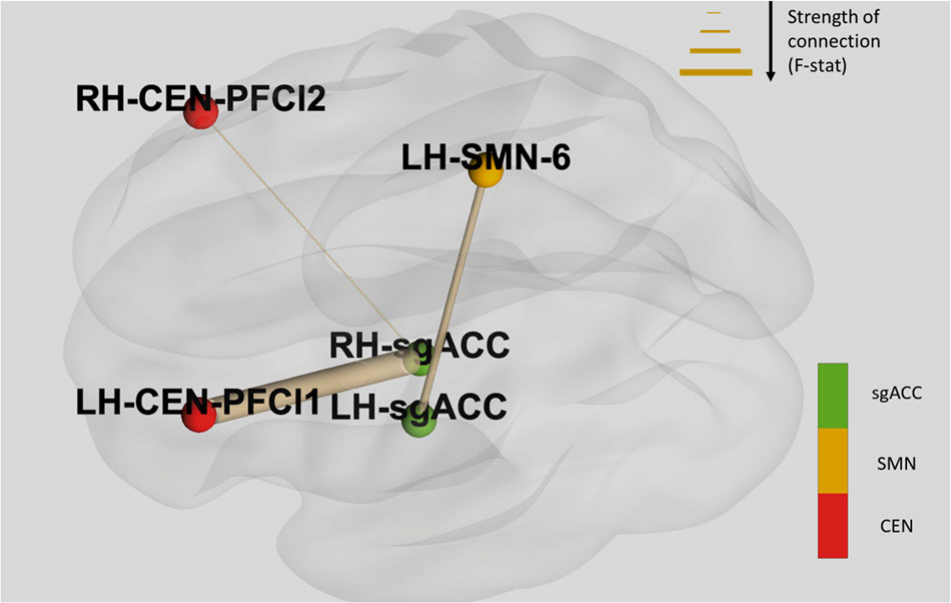
Changes in resting state functional connectivity between pre-and post-treatment which differed between groups with high vs low lassitude score at treatment day 3. Thickness of connections between nodes is determined by the F-statistic. Directionality of connectivity was as follows: left sgACC to LH_CEN_PFCl1- patients in the low group showed increased FC; left sgACC and RH_CEN_PFCl2- patients in the low group showed increased FC; right sgACC and LH_SMN_6- patients in the low group showed decreased FC. Notes: central executive network (CEN); sensorimotor network (SMN).

Three post treatment-pretreatment FC pair contrasts were found to be significantly different between groups which scored high and low on Lassitude at day 3. These pairs involved regions that were part of the central executive network (CEN) and sensorimotor network (SMN) and were: left sgACC to Left Hemisphere Central Executive Network lateral Prefrontal cortex area 1 where participants in the low group showed increased FC (F = 15.49, *p* = 0.001); left sgACC and Right Hemisphere Central Executive Network lateral Prefrontal cortex area 2 where participants in the low group showed increased FC (F = 8.68, *p* = 0.008); and, right sgACC and Left Hemisphere Sensorimotor Network area 6, where participants in the low group showed decreased FC (F = 10.49, *p* = 0.004). These connections are demonstrated in Fig. 3.

No functional connectivity results survived correction for multiple comparisons. In Supplementary Section H, we present the counts of participants who experienced early change in item 7 by treatment group.

## Discussion

In this study we have demonstrated that, rather than all symptoms showing a uniform change over time, there seems to be a temporal order in symptom change that is predictive of treatment outcome and different in those receiving active and sham SNT, suggesting that this difference is caused by treatment effects. More specifically, we observed that the earliest item to show differences between the active and sham groups is MADRS item 7 (lassitude), despite this symptom not being significantly different between groups at baseline. Lassitude showed an early reduction in the active group and remained significantly different between groups from day 2 of treatment until day 5. These findings are interesting in light of recent work that has demonstrated that rTMS may indeed have differential effects in different symptom dimensions^21^.

Lassitude, as defined in the MADRS, is “a difficulty getting started, or slowness initiating and performing everyday activities”^22^. As such, it is related to, but also distinct from, other commonly reported symptoms of MDD such as poor sleep, psychomotor retardation, low energy or anhedonia, all of which may also affect the ability to carry out tasks. Indeed, in related work authors have argued that perceived energy and fatigue are related but clinically and neurobiologically distinct states^23^, supporting a fine-grained approach to symptom interpretation when considering the mechanistic meaning of changes in specific symptoms. The significance of this early difference in lassitude, mechanistically, may be related to how it changes the experience of patients outside of treatment- those patients with decreased lassitude may start re-engaging in their daily routines, which in turn would have a therapeutic effect similar to that seen in the behavioral activation literature, wherein changes in behavior engenders later change in mood and other depressive symptoms^24,25^. We further note that differences in lassitude appeared between the active and sham groups *prior* to differences in sleep in our sample, suggesting that there is an increased sense of being able to complete daily tasks prior to any benefits to energy which would be conferred by improved sleep.

Evidence for the mechanistic relevance of early lower lassitude scores is present. Firstly, lassitude score at day 3 predicted treatment outcome across groups in terms of immediate response and response at four weeks post-treatment, even after controlling for baseline depression severity. In addition, 7 of 9 SNT responders immediately after treatment had early changes in lassitude, compared to only 1 of 3 SNT non-responders. As such, it seems reasonable to adopt as a hypothesis for future study that changes in lassitude may be an important part of the mechanism underlying treatment response in SNT. One alternative hypothesis which should be examined in a larger study is that early improvement in lassitude may be a nonspecific marker of improvement: of the 3 participants in the sham group who remitted at any time, all 3 experienced early changes in lassitude.

The question remains as to why SNT engenders this change in lassitude. One possibility is that early change in lassitude is a feature of any effective antidepressant treatment. While significant evidence exists that early changes in total symptom severity can predict treatment response^26^, there is relatively little evidence from treatment studies for the importance of early changes in specific symptoms. One study found that patients who remitted when treated with hypericum or fluoxetine had greater early reductions in the general somatic symptoms question of the Hamilton Depression Rating Scale (HAM-D)^27^. While not precisely the same as lassitude, the somatic symptoms question on the HAM-D does include the related notions of “loss of energy and fatigability” (Hamilton, 1960). A later analysis pooling a larger dataset from the same study found that remission in the whole sample was still associated with changes in general somatic symptoms; furthermore, when considering the treatment branches separately, general somatic symptom improvement was associated with placebo group remission^28^. As such it is possible that early changes in lassitude are a feature of effective antidepressant treatment in general, and potentially a key “nexus” point through which effective treatments act. However, SNT does produce improvement in depression symptoms more rapidly than other treatments, and in patients for whom other treatments have not been effective. As such, SNT may achieve improvement in lassitude more quickly due to its accelerated nature, or more effectively, due to its more precise imaging-based targeting; analyses of studies of SNT compared to other treatments with sufficiently dense early symptom sampling could help to resolve this question. Another possibility is that improved regulation of sgACC function, a downstream target of SNT, is responsible for early changes in lassitude. Indeed, one recent study found that the sgACC mediates the relationship between non-exercise activity (e.g., engaging in one’s daily routine) and perceived energy (which is likely related to lassitude)^29^. Another (not mutually exclusive) possibility is that the primary target (LDLPFC) is responsible for reductions in lassitude. Indeed, one study found that participants asked to perform a tiring finger tapping experiment and who were exposed to a static magnetic field over the LDLPFC had reduced slowing of their tapping rate^30^. In addition, a study using another non-invasive brain stimulation technique, transcranial direct current stimulation, in patients with multiple sclerosis found that LDLPFC treatment ameliorated fatigue, which is related (but not identical) to lassitude^31^. In addition, the DLPFC has been linked to integration of information about reward and to the driving of behavior through influence over mesocortical and mesolimbic dopaminergic systems^32^. As such there is evidence that both LDLPFC and sgACC may be relevant to circuits which regulate energy perception and motivated behavior, both of which are clearly related to the concept of lassitude. Another hypothesis worth considering in future work would be whether SNT belongs to a category of treatments with differential impact on the symptom dimension which includes lassitude and psychomotor retardation. Indeed, in ref. ^33^ the authors found that escitalopram improved an “observed mood” dimension of symptoms (which included clinician-rated depressed mood, anxiety, activity, and psychomotor changes) more than nortriptyline. As such, future work could seek to determine if different categories of treatments act through specific mechanisms, one of which may involve the improvement of lassitude/psychomotor retardation. SNT may be an example of this category of treatments, and may produce rapid improvement because it preferentially modulates neural circuits relevant to these symptoms as described above.

While the results presented here suggest that SNT may have an early effect on lassitude which leads to symptomatic improvement, it is important to note that some sham responders also experienced improvements in lassitude. Part of the reason for this may be the intensive nature of the SNT treatment, as it requires patients to present early to the clinic, experience multiple treatment sessions per day, and interact with study staff and clinicians. This experience may have an activating effect which may directly reduce patient perception of their own lassitude. Further studies including larger sham SNT arms could compare sham SNT to other treatments (e.g. treatment as usual) and may help to identify if, in a given timespan, the SNT process itself, regardless of stimulation, leads to more improvement in lassitude than other treatments.

rTMS does not only produce localized effects; rather, by stimulating one region it can have effects on interconnected brain networks^34^. As such, we analyzed functional connectivity between the sgACC, which is both a downstream target of SNT and a hub for networks like the default mode network^35^, and other regions associated with known brain networks. The first analysis considered pre-treatment FC, and we demonstrated that participants with low day 3 lassitude scores showed reduced FC between sgACC and regions in the VN and DAN, and increased FC with PFC7 in the DMN. Increased deactivation in the DMN during emotion processing has been shown to be predictive of antidepressant response^36^; given that SNT is targeted at LDLPFC regions which are anticorrelated with sgACC, it is possible that those whose sgACC is positively connected with the DMN at baseline are those who will benefit most from SNT. Hypoconnectivity of the DAN with frontal regions has also been found in MDD; as the DAN is involved in orienting to external stimuli, Improvements in DAN function may help people attend to the daily tasks they must accomplish, and in this way reduce lassitude^37^. VN hypoconnectivity is also seen in MDD and has been implicated in symptoms such as psychomotor retardation, which may again be relevant to lassitude^38^.

The second FC analysis was focused on FC changes between pre- and post-treatment. Participants with lower lassitude scores early in treatment showed increased FC between sgACC and regions in the CEN and reduced FC with a region in the SMN. Dysfunctional connectivity of the CEN has been implicated in MDD, with the assertion that its dysfunction negatively impacts goal directed behavior^39^; our results may suggest improved communication of the CEN with other brain regions, resulting in improvements in goal-directed behavior- which could reasonably be expected to manifest as reductions in lassitude. The SMN has also been implicated in MDD^40^, though increased FC between SMN and the fronto-parietal network (FPN) was seen in escitalopram responders^41^. As we only examined connectivity with sgACC, more complex connectivity patterns (e.g. with FPN) may explain this finding and could be examined in a larger sample.

If replicated, these FC patterns could serve as biomarkers to help identify which patients are most likely to experience changes in lassitude and treatment response, as well as to monitor response to treatment. Asking patients about changes in lassitude early in treatment may, if replicated, be proven a useful clinical marker of treatment response in accelerated rTMS, with patients who do not have early change in lassitude potentially being candidates for treatment modification. In addition, there may be synergistic benefits of combining rTMS with behavioral activation; indeed, studies have determined that combining rTMS and psychotherapy, including behavioral activation, is feasible, but there is a lack of evidence proving efficacy in randomized trials^42,43^.

Our most significant limitation is the small sample size which leads to our analyses being underpowered. Indeed, the preliminary results presented here would not survive correction for multiple comparisons, though we were able to partially replicate our clinical results in a previous open label dataset. As such, this work should be considered an exploratory, hypothesis generating analysis, the results of which must be replicated in subsequent studies. Future work could also expand on these results (e.g., in order to probe group by time interactions as well as to examine mediation and moderation). The use of only one assessment of symptom burden- the MADRS- is another limitation. Other scales capture other symptoms, and in larger samples with a more complete set of daily measures, additional early symptom differences may become apparent. Future work could also use questionnaires specifically adapted for more frequent administration (e.g. ref. ^44^). In addition, changes in other symptom categories not often measured by questionnaires but by tasks, such as cognition, may also be relevant early predictors of treatment response, and these are not considered here beyond the MADRS question on concentration^45^. Another limitation is our decision to limit our imaging analysis to only those pairs including the sgACC. While this was necessary given the small sample size of the study, it does potentially mean that some relevant FC pairs have been omitted from this analysis. In addition, due to the small sample size and exploratory nature of the analysis, we did not control for potentially important covariates (e.g., age, sex, and degree of treatment resistance) which will be important in future analyses. Better powered studies in the future should therefore examine a larger number of FC pairs as well as other imaging metrics.

In conclusion, we have identified that MADRS item 7 (lassitude) is the earliest symptom which differs between patients receiving SNT and sham treatment, as early as treatment day 2. In addition, the level of lassitude at treatment day 3 is a good predictor of treatment outcome, even when controlling for baseline depression severity. Resting state functional connectivity analyses suggest that connections between the sgACC, the ultimate target of LDLPFC-rTMS, and elements of the VN, DAN and DMN at baseline may predict lassitude at treatment day 3. In addition, changes in FC between sgACC and elements of the CEN and SMN may reflect early lassitude score and may represent biomarkers of response of this symptom to treatment. This exploratory analysis provides hypotheses which should be investigated in larger datasets.

## Supplementary information

Supplementary material

## Data Availability

Data used for this secondary analysis was provided by the authors of Cole et al., 2022. Inquiries regarding access to this dataset should be directed to N.R.W. (nolanw@stanford.edu).
