# Supplementary material for: Early differences in lassitude predicts outcomes in Stanford Neuromodulation Therapy for difficult to treat depression
Source: Npj Ment Health Res. 2024 Oct 28;3:49. doi: 10.1038/s44184-024-00099-2 (PMC11519532; doi:10.1038/s44184-024-00099-2)
Supplement: Supplementary file 1 — Supplementary material [file 44184_2024_99_MOESM1_ESM.pdf]

Supplementary material:

Supplementary Section A: Supplementary Results

Supplementary results for replication of results in the open label data from Cole et al. 2020:

Given that our analysis was exploratory and uncorrected, we sought to replicate it in another dataset. While no other SNT dataset with daily MADRS ratings was available, daily ratings during treatment using the HAMD-6 were available from our previous open label study of the SNT protocol (Cole et al., 2020). Although the HAMD-6 does not include a lassitude item, it does include a psychomotor retardation item. Lassitude has been described as the MADRS item most representative of psychomotor retardation [1], though the items do not fully overlap. There were 24 participants in this dataset with data available for the HAMD-6 and week 4 response on the MADRS; of these 14 (58.3%) were responders. Controlling for baseline MADRS total score, bootstrapped logistic regression analysis revealed that HAMD-6 psychomotor retardation score at day 3 was a predictor of response at 4 weeks post treatment ($B = -23.2$, $p = 0.005$; model Nagelkerke $R2 = 0.35$). This was also found at day 2 ($B = -2.3$, $p = 0.009$; model Nagelkerke $R2 = 0.41$; $n = 23$ subjects). This helps to increase confidence in our finding that early lassitude score predicts SNT outcome.

1. Buyukdura JS, McClintock SM, Croarkin PE. Psychomotor retardation in depression: biological underpinnings, measurement, and treatment. Prog Neuropsychopharmacol Biol Psychiatry. 2011 Mar 30;35(2):395-409. doi: 10.1016/j.pnpbp.2010.10.019. Epub 2010 Oct 31. PMID: 21044654; PMCID: PMC3646325.

Supplementary results for Cole et. al 2022 data:

Interestingly, after the acute treatment period, starting at week 1 post treatment, MADRS item 7 is no longer different between groups. Rather, other items- most consistently Apparent Sadness, Reported Sadness, Reduced Sleep and Inability to Feel, as well as the total score, are consistently different between groups. At day 3, within the active group, MADRS item 7 was the item most strongly correlated with MADRS total scores at week 4 (Supplementary Section C).

Full ANOVA Results for immediate post-treatment and 1 and 4 weeks post-treatment

These results do not appear in the main paper and are included for completeness.

For variables, MADRS_tot denotes MADRS total score and “MADRS1” or “MADRS_q1” denotes the numbered items. The suffix denotes the week (the suffix “fu” is for immediate post-treatment followup).

ANOVA at immediate post-treatment:

ANOVA

		Sum of Squares	df	Mean Square	F	Sig.
madrsfu	Between Groups	1589.858	1	1589.858	20.545	.000
	Within Groups	1625.098	21	77.386		
	Total	3214.957	22			
madrs1_fu	Between Groups	30.842	1	30.842	19.845	.000
	Within Groups	32.636	21	1.554		
	Total	63.478	22			
madrs2_fu	Between Groups	43.642	1	43.642	29.025	.000
	Within Groups	31.576	21	1.504		
	Total	75.217	22			
madrs3_fu	Between Groups	21.250	1	21.250	11.568	.003
	Within Groups	38.576	21	1.837		
	Total	59.826	22			
madrs4_fu	Between Groups	17.882	1	17.882	10.273	.004
	Within Groups	36.553	21	1.741		
	Total	54.435	22			
madrs5_fu	Between Groups	6.273	1	6.273	3.906	.061
	Within Groups	33.727	21	1.606		
	Total	40.000	22			
madrs6_fu	Between Groups	9.519	1	9.519	4.715	.041
	Within Groups	42.394	21	2.019		
	Total	51.913	22			
madrs7_fu	Between Groups	17.123	1	17.123	12.759	.002
	Within Groups	28.182	21	1.342		
	Total	45.304	22			
madrs8_fu	Between Groups	26.380	1	26.380	19.038	.000
	Within Groups	29.098	21	1.386		
	Total	55.478	22			
madrs9_fu	Between Groups	12.913	1	12.913	5.770	.026
	Within Groups	47.000	21	2.238		
	Total	59.913	22			
madrs10_fu	Between Groups	.174	1	.174	.123	.730
	Within Groups	29.826	21	1.420		
	Total	30.000	22			

ANOVA at 1 week post treatment:

ANOVA

		Sum of Squares	df	Mean Square	F	Sig.
madrs_tot_1wk	Between Groups	953.643	1	953.643	9.940	.006
	Within Groups	1630.989	17	95.941		
	Total	2584.632	18			
madrs1_1wk	Between Groups	16.115	1	16.115	6.744	.019
	Within Groups	40.622	17	2.390		
	Total	56.737	18			
madrs2_1wk	Between Groups	16.115	1	16.115	6.744	.019
	Within Groups	40.622	17	2.390		
	Total	56.737	18			
madrs3_1wk	Between Groups	4.950	1	4.950	1.950	.181
	Within Groups	43.156	17	2.539		
	Total	48.105	18			
madrs4_1wk	Between Groups	11.137	1	11.137	5.991	.026
	Within Groups	31.600	17	1.859		
	Total	42.737	18			
madrs5_1wk	Between Groups	4.529	1	4.529	3.639	.073
	Within Groups	21.156	17	1.244		
	Total	25.684	18			
madrs6_1wk	Between Groups	12.637	1	12.637	5.638	.030
	Within Groups	38.100	17	2.241		
	Total	50.737	18			
madrs7_1wk	Between Groups	6.571	1	6.571	3.496	.079
	Within Groups	31.956	17	1.880		
	Total	38.526	18			
madrs8_1wk	Between Groups	32.295	1	32.295	14.684	.001
	Within Groups	37.389	17	2.199		
	Total	69.684	18			
madrs9_1wk	Between Groups	7.734	1	7.734	2.265	.151
	Within Groups	58.056	17	3.415		
	Total	65.789	18			
madrs10_1wk	Between Groups	.337	1	.337	.551	.468
	Within Groups	10.400	17	.612		
	Total	10.737	18			

ANOVA at 2 weeks post treatment:

ANOVA

		Sum of Squares	df	Mean Square	F	Sig.
madrs_score_wk2	Between Groups	1009.406	1	1009.406	11.404	.003
	Within Groups	1681.736	19	88.512		
	Total	2691.143	20			
madrs_q1_wk2	Between Groups	15.464	1	15.464	9.030	.007
	Within Groups	32.536	19	1.712		
	Total	48.000	20			
madrs_q2_wk2	Between Groups	17.143	1	17.143	7.791	.012
	Within Groups	41.809	19	2.200		
	Total	58.952	20			
madrs_q3_wk2	Between Groups	8.606	1	8.606	4.034	.059
	Within Groups	40.536	19	2.133		
	Total	49.143	20			
madrs_q4_wk2	Between Groups	11.786	1	11.786	5.816	.026
	Within Groups	38.500	19	2.026		
	Total	50.286	20			
madrs_q5_wk2	Between Groups	5.626	1	5.626	2.277	.148
	Within Groups	46.945	19	2.471		
	Total	52.571	20			
madrs_q6_wk2	Between Groups	8.729	1	8.729	4.094	.057
	Within Groups	40.509	19	2.132		
	Total	49.238	20			
madrs_q7_wk2	Between Groups	6.656	1	6.656	4.135	.056
	Within Groups	30.582	19	1.610		
	Total	37.238	20			
madrs_q8_wk2	Between Groups	30.861	1	30.861	13.868	.001
	Within Groups	42.282	19	2.225		
	Total	73.143	20			
madrs_q9_wk2	Between Groups	17.490	1	17.490	6.770	.018
	Within Groups	49.082	19	2.583		
	Total	66.571	20			
madrs_q10_wk2	Between Groups	.097	1	.097	.123	.730
	Within Groups	15.045	19	.792		
	Total	15.143	20			

ANOVA at 4 weeks post treatment:

ANOVA

		Sum of Squares	df	Mean Square	F	Sig.
madrs_score_wk4	Between Groups	642.218	1	642.218	8.717	.008
	Within Groups	1399.782	19	73.673		
	Total	2042.000	20			
madrs_q1_wk4	Between Groups	12.364	1	12.364	7.978	.011
	Within Groups	29.445	19	1.550		
	Total	41.810	20			
madrs_q2_wk4	Between Groups	10.944	1	10.944	5.837	.026
	Within Groups	35.627	19	1.875		
	Total	46.571	20			
madrs_q3_wk4	Between Groups	8.485	1	8.485	7.988	.011
	Within Groups	20.182	19	1.062		
	Total	28.667	20			
madrs_q4_wk4	Between Groups	8.485	1	8.485	4.716	.043
	Within Groups	34.182	19	1.799		
	Total	42.667	20			
madrs_q5_wk4	Between Groups	.693	1	.693	.325	.576
	Within Groups	40.545	19	2.134		
	Total	41.238	20			
madrs_q6_wk4	Between Groups	7.093	1	7.093	2.920	.104
	Within Groups	46.145	19	2.429		
	Total	53.238	20			
madrs_q7_wk4	Between Groups	2.500	1	2.500	1.426	.247
	Within Groups	33.309	19	1.753		
	Total	35.810	20			
madrs_q8_wk4	Between Groups	22.111	1	22.111	7.908	.011
	Within Groups	53.127	19	2.796		
	Total	75.238	20			
madrs_q9_wk4	Between Groups	6.338	1	6.338	3.096	.095
	Within Groups	38.900	19	2.047		
	Total	45.238	20			
madrs_q10_wk4	Between Groups	.156	1	.156	.141	.712
	Within Groups	21.082	19	1.110		
	Total	21.238	20			

Supplementary Section B: Full binary logistic regression results when controlling for treatment group and baseline MADRS

MADRS_tot_b is the total baseline MADRS score. MADRS7_3 is the MADRS item 7 score on day 3. A1_S0 is the variable that codes for active vs. sham treatment.

Model Summary

Step	-2 Log likelihood	Cox & Snell R Square	Nagelkerke R Square
1	18.495[a]	.386	.532

a. Estimation terminated at iteration number 6 because parameter estimates changed by less than .001 for split file $bootstrap_split = 0.

Classification Table[a]

	Observed		Predicted: madrs_response_wk4_locf .00	Predicted: madrs_response_wk4_locf 1.00	Percentage Correct
Step 1	madrs_response_wk4_locf	.00	12	3	80.0
		1.00	2	6	75.0
	Overall Percentage				78.3

a. The cut value is .500

Variables in the Equation

		B	S.E.	Wald	df	Sig.	Exp(B)
Step 1[a]	madrs_tot_b	.225	.163	1.913	1	.167	1.253
	madrs7_3	-1.023	.792	1.667	1	.197	.360
	A1_S0	2.325	1.527	2.316	1	.128	10.224
	Constant	-7.209	5.581	1.668	1	.196	.001

a. Variable(s) entered on step 1: madrs_tot_b, madrs7_3, A1_S0.

Bootstrap for Variables in the Equation

		B	Bootstrap[a]: Bias	Bootstrap[a]: Std. Error	Bootstrap[a]: Sig. (2-tailed)	Bootstrap[a]: 95% Confidence Interval Lower	Bootstrap[a]: 95% Confidence Interval Upper
Step 1	madrs_tot_b	.225	2.419[b]	9.131[b]	.057[b]	-.404[b]	31.630[b]
	madrs7_3	-1.023	-8.995[b]	31.404[b]	.024[b]	-109.818[b]	.606[b]
	A1_S0	2.325	20.112[b]	55.045[b]	.018[b]	.013[b]	196.915[b]
	Constant	-7.209	-76.561[b]	284.320[b]	.035[b]	-952.496[b]	27.201[b]

a. Unless otherwise noted, bootstrap results are based on 1000 bootstrap samples

b. Based on 997 samples

Supplementary Section C: Correlation between MADRS items at day 3 and total MADRS at 4 weeks post treatment amongst active groups patients (*= significant at p=0.05; **=significant at p=0.01):

Correlations

		madrs_wk4_locf	madrs_tot_3	madrs1_3	madrs2_3	madrs3_3	madrs4_3	madrs5_3	madrs6_3	madrs7_3	madrs8_3	madrs9_3	madrs10_3
madrs_wk4_locf	Pearson Correlation	1	.232	.032	.175	.116	-.273	-.447	.585*	.737**	.561	.264	-.004
	Sig. (2-tailed)		.467	.922	.586	.719	.391	.145	.046	.006	.058	.407	.990
	N	12	12	12	12	12	12	12	12	12	12	12	12

Supplementary Section D: Supplementary introduction and discussion

Supplementary introduction:

While antidepressants are certainly effective in managing a major depressive episode (Cipriani et al., 2018), they may have side effects which can lead to non-adherence or to reduced quality of life (Bet et al., 2013; Kelly et al., 2008; Lingam & Scott, 2002). As such, treatments for TRD which do not carry the same risk of side effects over time would be ideal. Even if patients need to remain on medication to prevent relapse, avoiding the need to add a new drug to a patient's current pharmacological regimen while providing relief from depressive symptoms would be clinically useful.

A commonly used FDA approved protocol for rTMS sees patients come in for a single daily session of rTMS over 6 weeks of treatment (Perera et al., 2016). Originally, this 6 week treatment protocol utilized a 37.5 minute long single daily session of rTMS. However, the FDA in 2018 approved a new version of rTMS called intermittent theta burst stimulation (iTBS) (Cohen et al., 2022). iTBS delivers pulses more rapidly, and each session now only takes approximately 3 minutes. This recent FDA approval came after the results of a non-inferiority trial reporting that iTBS was non-inferior to 10Hz of rTMS in the treatment of MDD (Blumberger et al., 2018). Not only did this trial demonstrate non-inferior efficacy, but also very similar safety, tolerability, and acceptability profiles. The primary advantage of iTBS over traditional rTMS is, of course, that the treatment takes 1/10th of the time to deliver, increasing the number of patients who can be treated. However, patients must still commit to six weeks of treatment, meaning that many individuals may need to travel or miss work in order to attend sessions. As such, shortening the time required for the antidepressant effect of rTMS would be an ideal way to improve its clinical utility.

Supplementary discussion:

From a mechanistic standpoint, it is interesting to consider that between-group differences in lassitude are only apparent acutely during treatment. This may suggest that improvement in lassitude may have some important permissive role- that it allows other changes to take place, which in turn lead to improvement in the depressed phenotype. While detailed mediation analyses were not possible given the sample size in this study, they could be carried out in larger studies.

Supplementary section E: Bootstrapped binary logistic regression using MADRS item 7 score predicting outcome at 4 weeks post treatment and controlling for MADRS total at baseline.

Using item 7 score at Day 2 as the predictor, nagelkerke R2 was 0.28 and classification accuracy was 87%. Without bootstrapping, neither MADRS item 7 at Day 2 or MADRS total were predictive of outcome. With bootstrapping, MADRS item 7 at day 2 was predictive of outcome (B = -0.85, p = 0.037) and MADRS total was not predictive.

Supplementary Section E: Full binary logistic regression results for MADRS7 at day 3 predicting response, controlling for baseline MADRS. MADRS_tot_b is the total baseline MADRS score. MADRS7_3 is the MADRS item 7 score on day 3. MADRS_response_wk4_locf is the variable that codes for response (binary) at 4 weeks post treatment, with LOCF used to carry forward results for two patients who were not observed at 4 weeks post-treatment.

Model Summary

Step	-2 Log likelihood	Cox & Snell R Square	Nagelkerke R Square
1	21.437[a]	.302	.417

a. Estimation terminated at iteration number 5 because parameter estimates changed by less than .001 for split file $bootstrap_split = 0.

Classification Table[a]

Observed			Predicted madrs_response_wk4_locf .00	Predicted madrs_response_wk4_locf 1.00	Percentage Correct
Step 1	madrs_response_wk4_locf	.00	14	1	93.3
		1.00	4	4	50.0
	Overall Percentage				78.3

a. The cut value is .500

Bootstrap for Variables in the Equation

		B	Bootstrap[a] Bias	Std. Error	Sig. (2-tailed)	95% Confidence Interval Lower	Upper
Step 1	madrs7_3	-1.545	-4.342[b]	21.236[b]	.005[b]	-53.570[b]	-.691[b]
	madrs_tot_b	.182	.766[b]	5.702[b]	.122[b]	-.120[b]	8.256[b]
	Constant	-3.356	-17.681[b]	175.763[b]	.363[b]	-157.246[b]	38.204[b]

a. Unless otherwise noted, bootstrap results are based on 1000 bootstrap samples

b. Based on 999 samples

Variables in the Equation

		B	S.E.	Wald	df	Sig.	Exp(B)
Step 1[a]	madrs7_3	-1.545	.778	3.949	1	.047	.213
	madrs_tot_b	.182	.141	1.677	1	.195	1.200
	Constant	-3.356	3.935	.727	1	.394	.035

a. Variable(s) entered on step 1: madrs7_3, madrs_tot_b.

Null model:

Classification Table[a,b]

	Observed		Predicted madrs_response_wk4_locf .00	1.00	Percentage Correct
Step 0	madrs_response_wk4_locf	.00	15	0	100.0
		1.00	8	0	.0
	Overall Percentage				65.2

a. Constant is included in the model.

b. The cut value is .500

Supplementary section F: Correlation of daily MADRS and BDI-II ratings

Daily MADRS ratings showed reasonable correlations with self-reported symptoms (as measured by the Beck Depression Index (BDI-II) (Beck et al., 1996) as of the second day of treatment: correlation at baseline 0.21; day 1 0.36; day 2 0.56**; day 3 0.69**; day 4 0.76**; day 5 0.77**; week 4 0.79**

Supplementary Section G: MADRS item 7 over time in responders vs. non-responders, and of active vs. sham for just treatment days (expanded exploratory analysis):

In addition, MADRS item 7 was different over time between responders and non-responders, with responders at week 4 post-treatment showing early reduction in and overall lower MADRS item 7 scores, even when correcting for MADRS total at baseline (Figure 2; repeated measures GLM, F(1)=15.6, *p*= 0.01). N = 18 due to missing data.

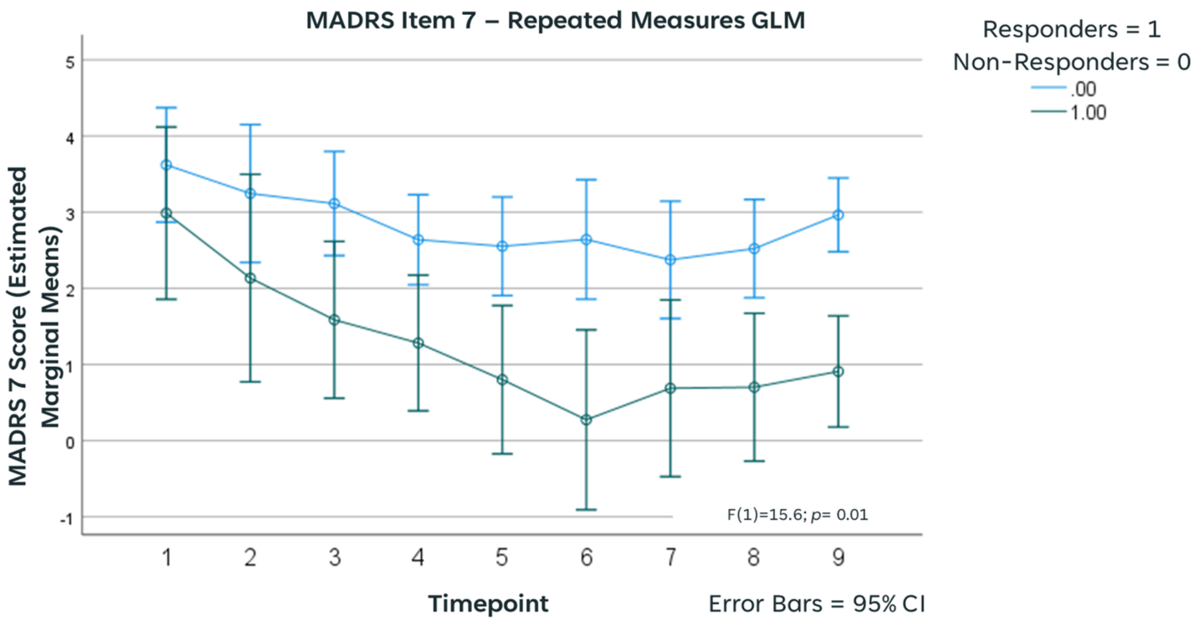

Repeated Measures GLM of MADRS Item 7 Scores, main effect of group between Responders and Non-Responders at week 4 post-treatment, controlling for baseline MADRS score. MADRS 7 score is in estimated marginal means. Timepoints: 1 = baseline; 2-6 = treatment days; 7 = 1 week post treatment; 8 = 2 weeks post treatment; 9 = 4 weeks post treatment

Transformed Variable: Average

Source	Type III Sum of Squares	df	Mean Square	F	Sig.	Partial Eta Squared
Intercept	871.336	1	871.336	178.333	.000	.895
A1_S0	36.553	1	36.553	7.481	.012	.263
Error	102.606	21	4.886			

Profile Plots

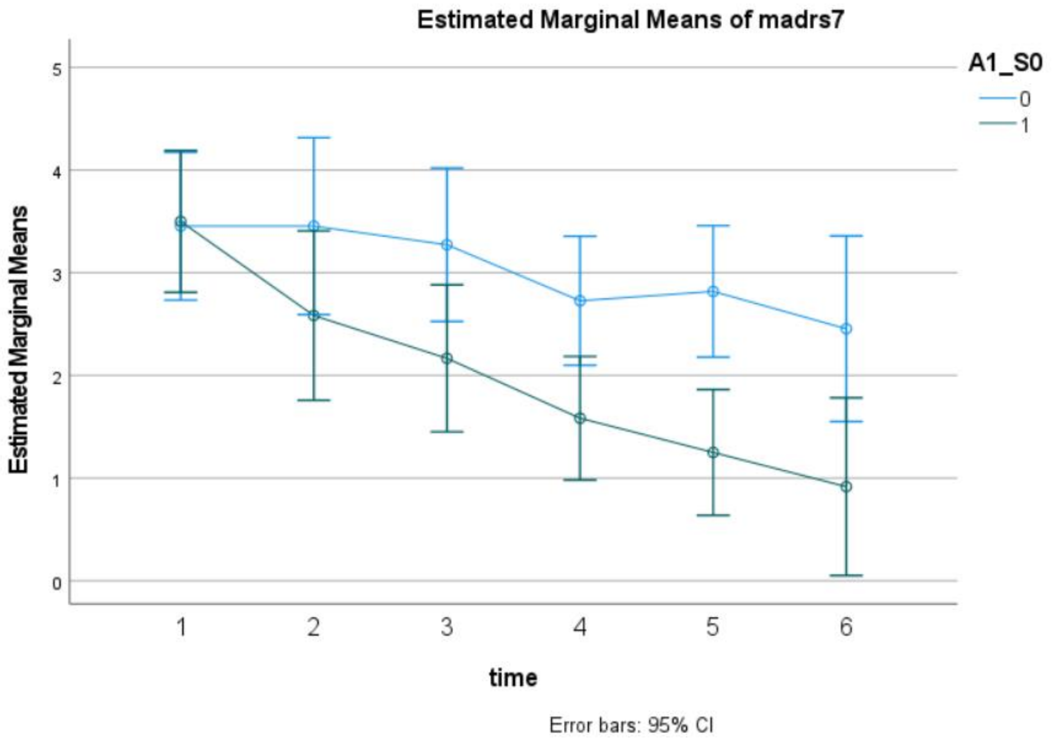

Repeated Measures GLM of MADRS Item 7 Scores between Active and Sham groups for only baseline and the treatment days; main effect of group. MADRS 7 score is in estimated marginal means. Timepoints: 1 = baseline; 2-6 = treatment days. A1= Active = 1; S0; Sham = 0.

Here with n = 23 (no missing data) we see that MADRS7 is still different between active and sham groups ($F(1) = 7.48$, $p = 0.012$).

In addition, no other MADRS items had significantly different main effects between groups when examined using GLM and examining only baseline and treatment days. However, MADRS 10 (suicidality) and MADRS 2 (apparent sadness) did have significant group x time interactions (MADRS 10 ($F = 2.8$, $p = 0.02$), MADRS 2 ($F=3.6$, p (Greenhouse-Geisser corrected 0.03). Active vs. sham comparisons are available in the figures below; MADRS 10 score is difficult to interpret given apparent baseline differences

in suicidality. MADRS 2 scores seem to differ between groups as of treatment day 3, but this is not significant in the ANOVA analyses at each day. These results bear further investigation in future work.

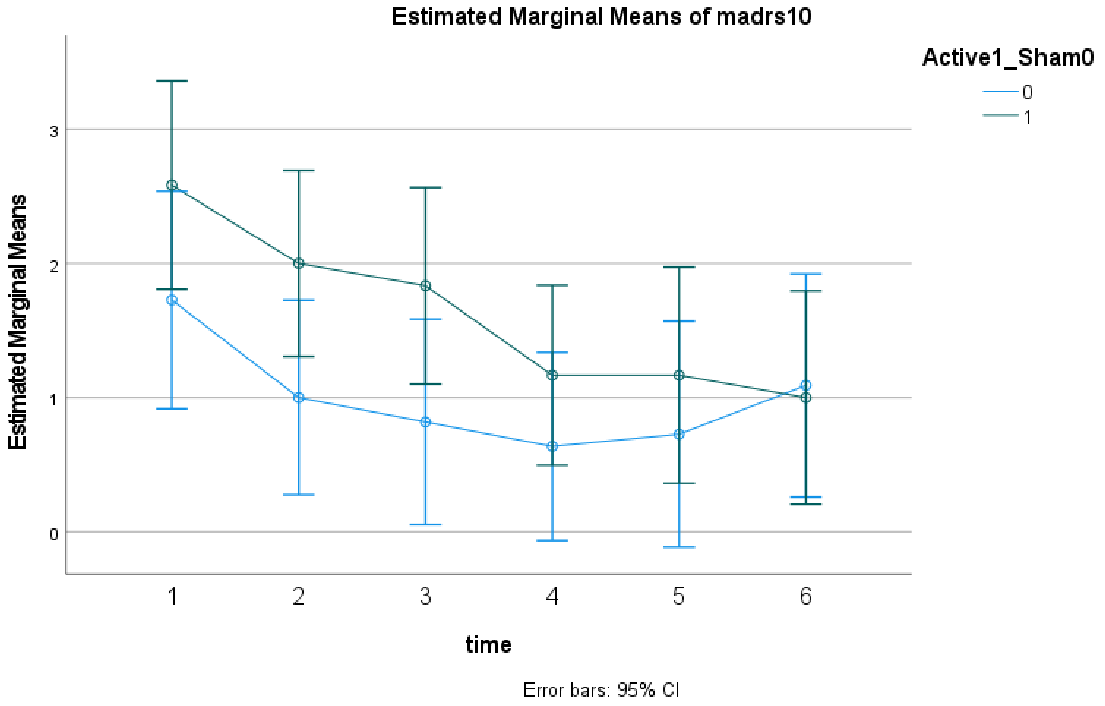

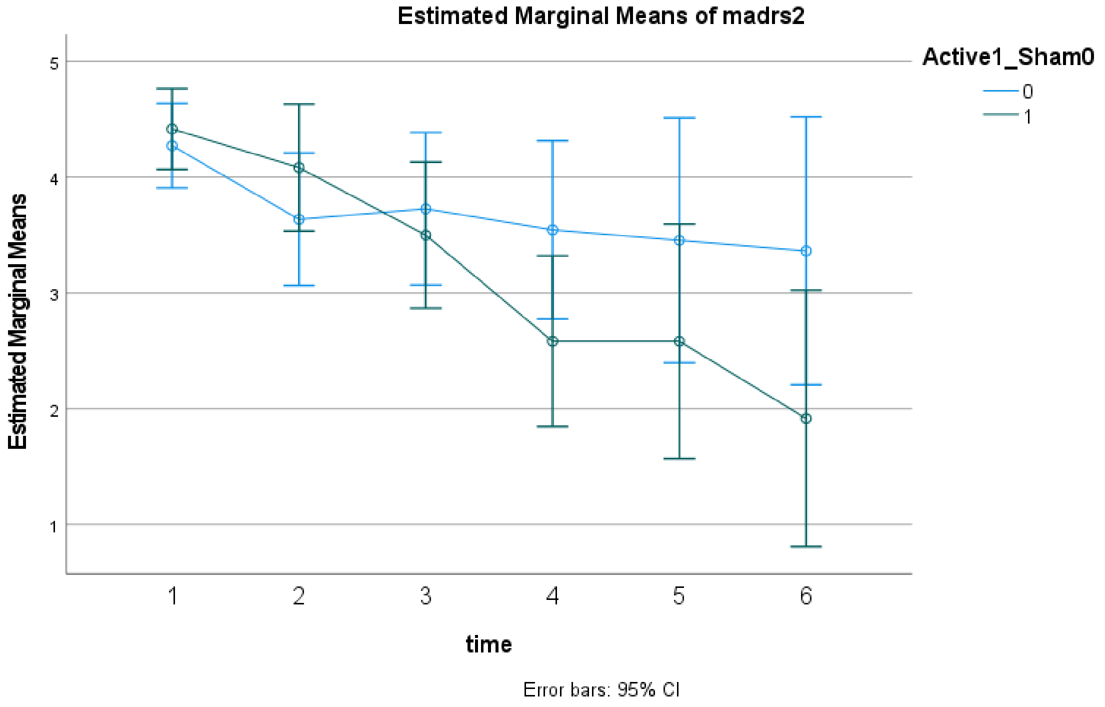

Repeated Measures GLM of MADRS Item 2 and 10 Scores between Active and Sham groups for only baseline and the treatment days; main effect of group. MADRS 2 and 10 scores are in estimated marginal means. Timepoints: 1 = baseline; 2-6 = treatment days. A1= Active = 1; S0; Sham = 0.

In addition, if we restrict the analysis to solely baseline compared to day 3, there is a group by time interaction (F = 4.6, p = 0.04). However the between-subjects effect is no longer significant (p = 0.14).

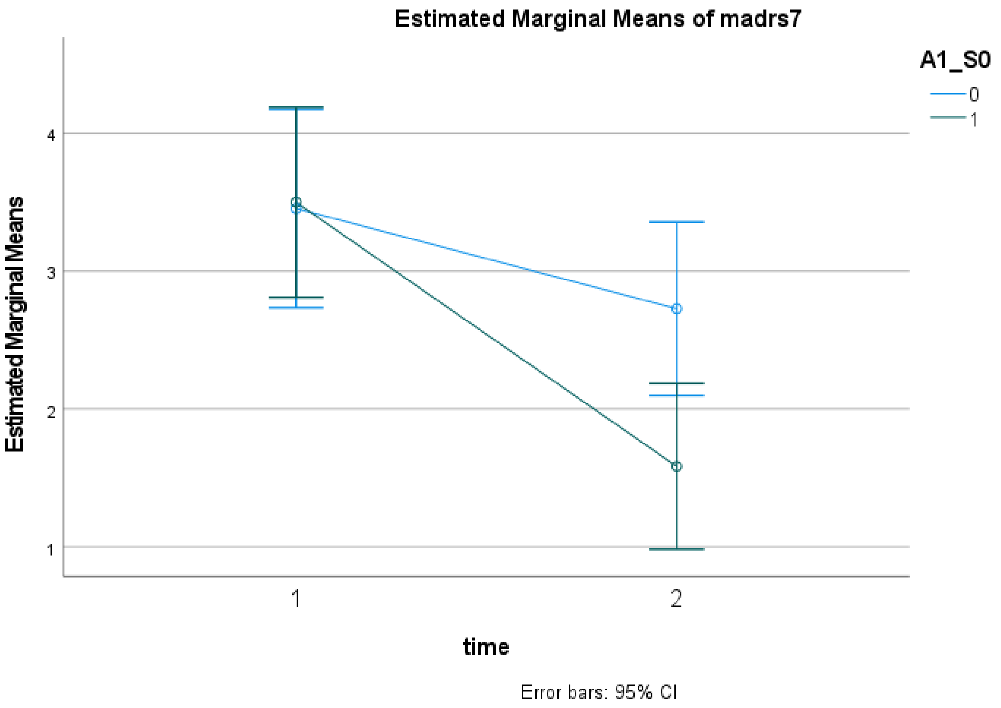

Supplementary Section H: Count of patients experiencing early change in lassitude by treatment condition and response/remission

Outcome definition	SNT Response/Remission	SNT non-response/remission	Sham response/remission	Sham non-response/remission
Response at 4 weeks post treatment	4/7	4/5	1/1	2/10

Response immediately after treatment	7/9	1/3	2/2	1/9
Remission at any time	7/10	1/2	3/3	0/8

Table: Proportion of patients per outcome definition who had an early change in lassitude at day 3 (score of 2 or less). E.g. in the upper left most cell, 4 of 7 patients in the active group who experienced response also had a day 2 lassitude score of 2 or less.

Supplementary section I: Remission Analysis

We examined whether lassitude score at day 3, controlled for baseline MADRS, could predict remission at any time; remission was defined as < 11 points on the MADRS [1.2]. Similar to other analyses, day 3 lassitude did predict remission at any time: (model: *X2*(2) = 14.9; *p* = 0.001; lassitude (B = -2.8, *p* = 0.003, 95% CI: [-129.3, -1.67]); the model had a classification accuracy of 82.6% and nagelkerke R^2 of 0.64. Analysis of sustained remission, e.g. those experiencing remission at the end of the four weeks of treatment, was not possible as only 4 patients remained in remission at that timepoint.

1. Kaneriya, S. H. et al. Predictors and Moderators of Remission With Aripiprazole Augmentation in Treatment-Resistant Late-Life Depression: An Analysis of the IRL-GRey Randomized Clinical Trial. JAMA Psychiatry 73, 329–336
2. Carstens L, Hartling C, Stippl A, Domke AK, Herrera-Mendelez AL, Aust S, Gärtner M, Bajbouj M, Grimm S. A symptom-based approach in predicting ECT outcome in depressed patients employing MADRS single items. Eur Arch Psychiatry Clin Neurosci. 2021 Oct;271(7):1275-1284. doi: 10.1007/s00406-021-01301-8. Epub 2021 Jul 16. PMID: 34269881; PMCID: PMC8429160.
